# Low-Cost Platform for Multiplexed Electrochemical Melting Curve Analysis

**DOI:** 10.1021/acsmeasuresciau.1c00044

**Published:** 2021-11-22

**Authors:** Nassif Chahin, Santiago Escobar-Nassar, Johann Osma, Abdulaziz S. Bashammakh, Abdulrahman O. AlYoubi, Mayreli Ortiz, Ciara K. O’Sullivan

**Affiliations:** † Departament d’Enginyeria Química, Universitat Rovira i Virgili, Avinguda Països Catalans 26, 43007 Tarragona, Spain; ‡ Department of Electrical and Electronics Engineering, Universidad de los Andes, Cra. 1E No. 19a-40, Bogotá, DC 111711, Colombia; § Department of Chemistry, Faculty of Science, King Abdulaziz University, 21589 Jeddah, Kingdom of Saudi Arabia; ∥ ICREA, Passeig Lluis Companys 23, 08010 Barcelona, Spain

**Keywords:** electrochemical melting curve analysis, SNP detection, voltammetric measurements

## Abstract

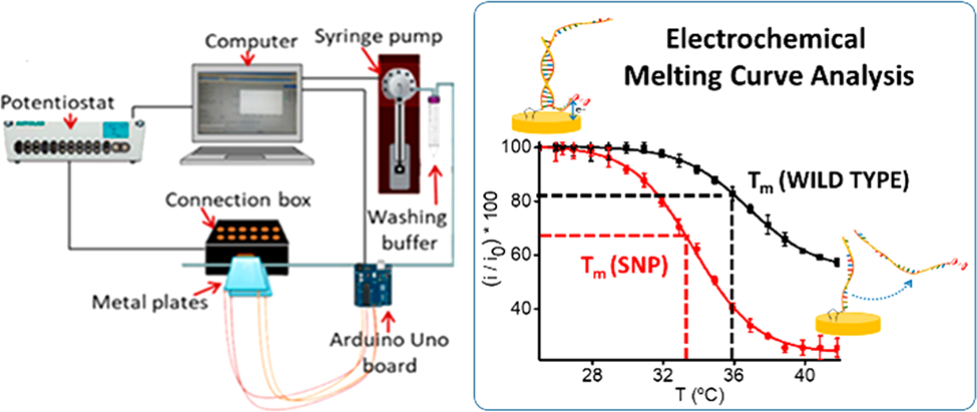

Detection and identification of single nucleotide polymorphisms (SNPs) have garnered increasing interest in the past decade, finding potential application in detection of antibiotic resistance, advanced forensic science, as well as clinical diagnostics and prognostics, moving toward the realization of personalized medicine. Many different techniques have been developed for genotyping SNPs, and ideally these techniques should be rapid, easy-to-use, cost-effective, flexible, scalable, easily automated, and requiring minimal end-user intervention. While high-resolution melting curve analysis has been widely used for the detection of SNPs, fluorescence detection does not meet many of the desired requirements, and electrochemical detection is an attractive alternative due to its high sensitivity, simplicity, cost-effectiveness, and compatibility with microfabrication. Herein, we describe the multiplexed electrochemical melting curve analysis of duplex surfaces tethered to electrodes of an array. In this approach, thiolated probes designed to hybridize to a DNA sequence containing the SNP to be interrogated are immobilized on gold electrodes. Asymmetric PCR using a ferrocene-labeled forward primer is used to generate this single-stranded redox-labeled PCR amplicon. Following hybridization with the probe immobilized on the electrode surface, the electrode array is exposed to a controlled ramping of temperature, with concomitant constant washing of the electrode array surface while simultaneously carrying out voltammetric measurements. The optimum position of the site complementary to the SNP site in the immobilized probe to achieve maximum differentiation in melting temperature between wild-type and single base mismatch, thus facilitating allelic discrimination, was determined and applied to the detection of a cardiomyopathy associated SNP.

## Introduction

Human genomes are 99.9% identical. Even so, a person hosts millions of variations in their gene coding regions, and the most common variations are due to single nucleotide polymorphisms (SNPs). These are single nucleotide variations in a defined genetic location and occur at a frequency of between 1 in 100 to 1 in 300 bases.^1,2^ Understanding of the importance and application of SNPs is an emerging field, and it is widely believed that SNPs will have a critical role in pharmacogenetics, disease genetics, and advanced forensics.^3^ As an increasing number of SNPs are identified using next-generation sequencing technologies, a battery of genotyping technologies for the detection of SNPs, including primer extension, ligation, enzymatic cleavage, mass spectroscopy, and conformational analysis, among others, have emerged.^4,5^ However, many of these approaches are expensive and labor-intensive and often require considerable infrastructure and instrumentation.

Hybridization approaches for the identification of SNPs exploit the differences in the thermal stability of double-stranded DNA between perfectly matched and mismatched target–probe pairs to achieve allelic discrimination.^6^ This methodology is referred to as melting curve analysis: a PCR amplified duplex is placed in a cuvette and the melting temperature is determined by measuring the UV–vis absorbance at 260 nm as the temperature is ramped using a Peltier. To improve the sensitivity of the technique, fluorescent intercalating dyes such as SBYR Green or Eva Green were employed, and the decrease in fluorescence with increasing temperature was measured. High-resolution melting curve analysis using highly controlled heating ramps and fluorescence detection facilitated single base mismatch differentiation. However, the technique is limited, as it cannot achieve high levels of multiplexing due to the limited availability of fluorescent intercalating with nonoverlapping emission spectra.^7^ Recently, a platform capable of multiplexed melting curve analysis was developed, where >1000 parallelized melts can be detected on a CMOS array. The platform, termed the Hydra 1K, uses cyanine labeled primers and asymmetric PCR to generate single-stranded labeled amplicons, which are captured via hybridization to probes immobilized on the CMOS array, which is then exposed to a controlled temperature ramp. The platform has been applied to the multiplexed fluorescence detection of 54 drug-resistance-associated mutations that are present in six genes of *Mycobacterium tuberculosis*.^8,9^

Electrochemistry is an attractive alternative to fluorescence due to its ease-of-use, high sensitivity, low cost, facile and cost-effective fabrication of electrode arrays, and compatibility with multiplexed detection with multichannel potentiostats. Indeed, different approaches of electrochemical melting curve analysis have been reported, using a variety of labels such as methylene blue, cobalt phenanthroline,^10,11^ cobalt bipyridine,^12,13^ ruthenium bipyridine,^14^ echinomycin,^15,16^ and epirubicin.^17^

Prest et al. developed a method using methylene blue as an intercalating redox molecule and measuring the change in the square wave voltammetry response to calculate the melting temperature.^18^ Defever et al. reported a real-time PCR and melting curve analysis method based on the use of a osmium bipyridyl complex ([Os(bpy)_2_ dppz]^2+^) intercalating redox molecule, again measuring the change in the square wave voltammetry response as the temperature was increased, and then generating the positive first-derivative analyses (d*i*/d*T*) of the melt.^19^ A microfluidic device integrating thermal control and a multielectrode array was designed by Shen et al., who performed a rapid electrochemical melting curve analyses on small-volume samples of 10 μL. In this case, an immobilized probe was hybridized to targets labeled with methylene blue and the dissociation of the labeled target measured using square wave voltammetry.^20^ In a similar approach, dsDNA denaturation between a ferrocene-labeled-PNA and a fully complementary or single-base-mismatched DNA on the negatively charged electrode surface of indium tin oxide (ITO) was monitored electrochemically.^21^

Nasef et al. described a method based on label-less electrochemical melting curve analysis for the detection of the cystic fibrosis associated DF508 mutant, which a 3-base deletion mutation. A 21-base thiolated probe was immobilized on a gold electrode and hybridized to a ssDNA PCR amplicons of mutant target (85 bases) or wild-type target of 82 bases. Methylene blue was used as an electrochemical indicator of hybridization, and differential pulse voltammograms were recorded during a discontinuous ramping of the temperature, and a clear discrimination between the melting temperature of the mutant and wild-type target was observed.^22^ Nasef et al. went on to report an alternative approach, using ferrocene labeled target, and in this approach two different electrodes of an array were functionalized with a probe complementary to the mutant and a probe complementary to the wild-type, and both were hybridized with the ferrocene-modified mutant sequence. The entire sensor array was subjected to discontinuous temperature ramping, and the dissociation of the ferrocene-labeled DNA from immobilized probes was monitored using DPV. The *T*_m_ recorded for the fully complementary and mismatched duplex was 38 and 30 °C, respectively, which provided a clear discrimination between matched and mismatched targets.^23^

The work reported herein aims to improve on these last two works, which were carried by Nasef et al. in our group. These previous works were very laborious, and while the use of electrochemical melting curve analysis for the detection of the DF508 was clearly demonstrated, the methodology required extensive hands-on time. In these previous works, the electrode was placed in a cuvette, which was housed in a Peltier device, and the temperature was increased in steps of 1 or 5 °C, and after each step, the electrode was removed from the cuvette and washed, and then electrochemical measurement was carried out in an electrochemical cell, and the electrode was returned to the cuvette for the next increase in temperature. In the work reported herein, we wanted to move toward a more automated setup, where the electrode array was housed in a microfluidic chamber, integrated within a Peltier heating device which could be programmed to ramp the temperature as desired. During the temperature ramp, the electrode array was continuously washed to remove denatured DNA, and the electrochemical signal was measured throughout.

**Figure 1:**
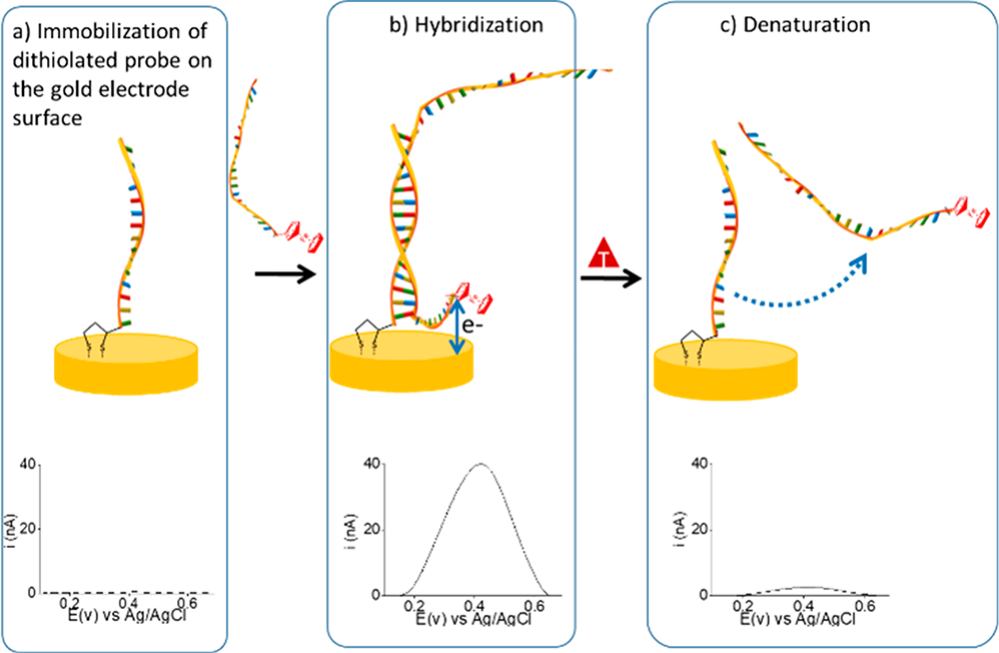
Conceptual schematic representation of the electrochemical melting curve analysis approach: (a) Immobilization of dithiolated probe on the gold electrode surface. (b) Hybridization with single-stranded DNA PCR amplicon containing the SNP to be interrogated. (c) Temperature ramping and denaturation of the surface-tethered duplex and concomitant decrease in the electrochemical signal.

The objective of this work was thus to develop a Peltier heating device using a computer-controlled heating block, consisting of a pair of aluminum blocks that can be heated in a controlled way using a pulse-width modulation protocol implemented on an Arduino UNO. Using this “in-house” semi-automated Peltier heating setup, we wanted to demonstrate that it could be used for carrying out multiplexed electrochemical melting curve analysis (éMCA) for the detection of a SNP associated with cardiomyopathy as a model system. To carry out a demonstration of a proof-of-concept of the éMCA, individual gold electrodes of an array were functionalized with thiolated 21-mer probes, which were designed to hybridize to a ferrocene-labeled complementary 21-mer oligo and subjected to a temperature ramp (Figure 1). As we wanted to explore if the position of the complementary to the SNP site on the immobilized probe had an effect on increasing the difference in melting temperature between fully complementary and mismatch, we design the probes to be fully complementary, or to have a mismatch at the top, bottom, or middle of the probe. Single-stranded ferrocene-labeled PCR 124-mer amplicons were also hybridized to the four different probes, as well as four different probes hybridized to the same 124-mer amplicon and subjected to éMCA, and finally, the results were confirmed using fluorescence melting curve analysis.

## Experimental Section

### Chemicals and Materials

Solutions were prepared using a Milli-Q water purifier system (Milipore, Madrid, Spain) with a resistance level of 18.2 MΩ cm. All chemicals and reagents were of analytical grade and used without further purification. Potassium dihydrogen phosphate (KH_2_PO_4_), acetone, and isopropanol were purchased from Scharlau, Spain. Potassium hydroxide (KOH), hydrogen peroxide (H_2_O_2_ 30% (v/v)), potassium ferricyanide (K_3_[Fe(CN)_6_]), and Tris buffer at 7.4 pH were provided by Sigma-Aldrich, Tres Cantos, Spain). Tris buffer pH was adjusted to 7.4 using 1 M HCl and 1 M NaOH (Sigma-Aldrich). The surface spacer dithiol 16-(3,5-bis((6-mercaptohexyl)oxy)phenyl)-3,6,9,12,15-pentaoxahexa-decane) (DT1)^24^ was obtained from SensoPath Technologies (Bozeman, MT, USA). Three-millimeter-thick poly(methyl methacrylate) (PMMA) was purchased from La Industria de la Goma (Tarragona, Spain) and the double-sided adhesive foil ARsealTM 90880 was purchased from Adhesive Research, Ireland. Oligonucleotide capture probes and targets were purchased as lyophilized powder from Biomers.net, Ulm, Germany), reconstituted in nuclease-free water (ThermoFisher Scientific, Spain) and used without further purification. Table S-1 (SI) shows the oligonucleotides sequences used in this study. Eva Green dye was purchased from Applied Biosystems (Spain), GelRed Nucleic Acid Gel Stain from Biotium (Barcelona, Spain), and DreamTaq DNA polymerase, Lambda exonuclease, and the certified molecular biology agarose gel powder from ThermoFisher Scientific (Spain).

### Melting Curve Analysis Device Components

The Arduino UNO, IRF520, the resistances, condensers, type K thermocouples, connectors, BI BPC10 resistors, AD595, breadboard, and the 2.5 W/mK thermally conductive tapes were all purchased from Farnell (Madrid, Spain). For the temperature reference system, a type K thermocouple connected to the precision thermometer Hi 93531 (Hanna instruments, Bilbao, Spain) was used. The variable DC power supply PeakTech 6006D (Telonic instruments LTD, Berkshire, UK) was used to supply the temperature reading system at 5.1 V.

**Figure 2:**
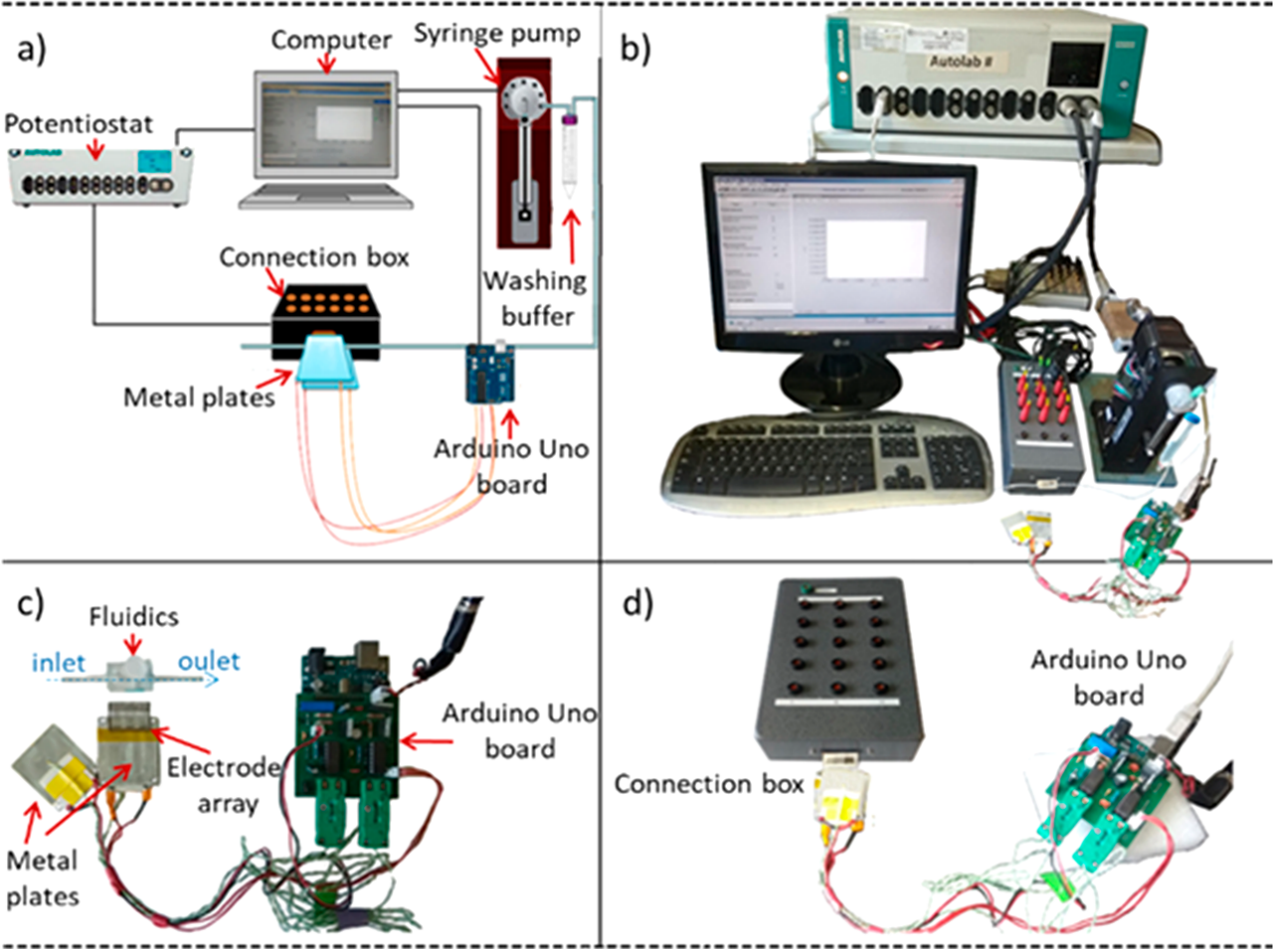
Setup of the integrated system for SNPs detection using éMCA: (a) Schematic of the system showing how the heating plates of a homemade Peltier surround the gold electrode array connected to the potentiostat, and the connection of the syringe pump propulsed washing buffer to the reaction chamber, where electrochemical measurements and temperature control take place. (b) Real picture of the whole setup of the éMCA system. (c,d) Homemade Peltier device consisting of an Arduino Uno board and two heating metal plates.

Figure 2a shows a schematic of an integrated system for the detection of SNPs, based on performing an electrochemical melting curve analysis using a homemade Peltier device. A gold electrode array functionalized with DNA is set between two aluminum plates of a homemade Peltier device providing a robust control and ramping of temperature (1 °C/Step). The Peltier consists of an Arduino Uno board using the free Arduino software for data visualization. The gold electrode array is covered by a poly(methyl methacrylate) (PMMA) microfluidic that allows liquid buffer to continuously wash the electrode array surface while heating.

### Methodology

#### Heating System Characterization

##### Transfer Function

A type-k thermocouple and a BI BPC10 680 J resistor were attached using conductive tape to each of the heating blocks. To determine the step response of each system, a 0.48 mV step was applied to each resistor. All the temperature readings were recorded with the Arduino and the Matlab 2013a software; the system behavior was modeled. An inspection of each response revealed that both corresponded to a type I system. Using Matlab, the process gain *K*_p_ and *t*_1_ and *t*_2_, which correspond to the time when the output attains the 63.2% and 28.3% of its final value, respectively, were defined. The theta (θ), tau (τ), and type I plus dead time-continuous transfer function can be calculated as follows:
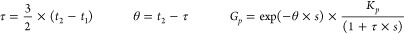


##### PID Tuning

The Matlab tool Simulink was used to model the closed-loop response of each PI controller in series with a heating plate. The correct model of the system behavior required the transfer function of each plate. We were able to tune and observe the closed-loop response and reference tracking of each system as well as the error, the controller effort, and the open-loop response, plus many other options using the Simulink tool PID tuner. For the PI control system, the following equation was used:
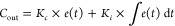
where *e*(*t*) represents the error or, the same, the set point input, *C*_out_ is the controller output, *K*_c_ is the proportional gain, and *K*_i_ represents the integral gain.

To validate the functionality of the system, a glass slide was used and covered by a microfluidic PMMA filled with buffer to simulate an electrode array. A thermocouple was glued inside the microfluidic to monitor the temperature. This setup was placed between the metal blocks (and a plastic case surrounded the plate to isolate it from the environment). The temperature was ramped and recorded by Arduino and compared with the one measured by the thermocouple. One end of the BI BPC power resistors was connected to a 24 V DC adapter and the other end to the drain of an IRF520 power transistor. These transistors are responsible for delivering a controlled current to each of the resistors. The power delivered by each IRF was modulated through the duty cycle variation of a 10 bit pulse width modulation (PWM) system implemented on the Arduino UNO. The PWM worked at a frequency of ∼4 kHz at 5 V. Finally, the glass slide and reference readings were recorded using the Hi 93531.

##### Electrode Fabrication

**Figure 3:**
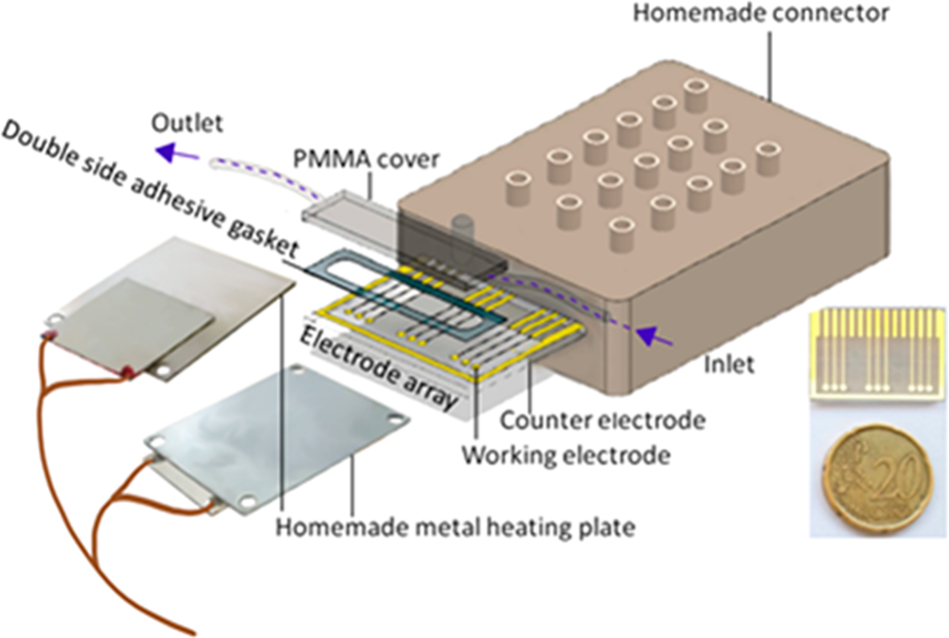
Schematic of the system setup where the electrode array is placed between two metal plates and covered by a microfluidic cell. The electrode array, double adhesive gasket, and PMMA cover are bound together to create 672 μL cells where hybridization and electrochemical measurements are carried out. The electrode array is heated by the aluminum heating plates from room temperature to 95 °C and is connected to the potentiostat through a homemade connector box.

The gold electrode array (Figure 3) was designed with nine circular working electrodes (1 mm^2^) and a rectangular counter electrode (4 mm^2^). It was fabricated in a clean room using 75 × 25 mm^2^ soda-lime glass slide substrate (Sigma-Aldrich, Spain). The sputtering processes consists of the following steps: after cleaning, the glass slides were subjected to an oxygen plasma etching using AC O_2_/Ar (5 cm^3^/s^–1^ of Ar, 5 cm^3^/s^–1^ of O_2_, 50 W) for 5 min. A positive photoresist AZ 1505 (Micro Chemicals GmbH, Germany) was deposited by spin coating at (4000 rpm for 30 s) on a precleaned and dried glass slide. The glass slide was then exposed to UV light for 4 s using a chromium mask in contact mode (LED Paffrath GmbH, Rose Foto Masken, Germany) and then immersed for 1 min in a commercial developer AZ 726. Following development, the glass slide was introduced into the sputtering chamber (ATC Orion 8-HV, AJA International Inc., USA) and was subjected to an oxygen plasma etching using AC O_2_/Ar (5 cm^3^ s^–1^ of Ar, 5 cm^3^ s^–1^ of O_2_, 50 W) for 5 min. Subsequently, a layer of 30 nm of Ti/TiO_2_ was sputtered (oxygen flow rate: 5 cm^3^ s^–1^ of O_2_ for the first 10 nm, then increased to 20 cm^3^ s^–1^ for the last 5 nm. (Ar flow rate: constant 5 cm^3^ s^–1^). The next step is the deposition of 100 nm of Au by AC sputtering (5 cm^3^ s^–1^ of Ar, 5 cm^3^ s^–1^ of O_2_, 50 W). Finally, the arrays were sonicated in acetone for 5 min, sonicated in isopropanol for 5 min, and then rinsed with Milli-Q water.

Custom-made microfluidics were fabricated using double adhesive gasket (Adhesive Research, Ireland) with 6-mm-thick PMMA cover plates patterned using a CO_2_ laser marker (Fenix, Synrad, USA). Following electrode array functionalization, a double adhesive gasket and PMMA were aligned and bonded to produce a 672 μL microfluidic chamber where DNA hybridization and electrochemical measurements were carried out. The washing buffer was flowed into the gold array through a tube embedded to the microfluidic chamber that was washed with 300 μL of (PBS pH 7.4) using a syringe pump (Cavro XL3000, Tecan Systems). Washing was driven by constant air pressure during melting curve analysis (Figure 3).

##### Electrode Functionalization

The electrode arrays were electrochemically cleaned by sweeping 10 times the potential from 0 to −1.2 V vs Ag/AgCl in 0.1 M deoxygenated aqueous KOH solution. After washing with Milli-Q water, the electrode surface was cleaned again by cycling the potential 10 times between 0.2 and 1.5 V in 0.5 M H_2_SO_4_. The gold electrode array were washed with Milli-Q water, dried with nitrogen, and used immediately for surface functionalization. Thiolated probe was self-assembled, via spotting of 1 μL of (1 μM probe solution), and DT1 as backfiller (ratio 1:100, thiolated probe:DT1) both freshly prepared in 1 M KH_2_PO_4_, onto each electrode surface of an array of 9 working electrodes, and then left to self-assemble for 3 h in a humidity chamber to prevent evaporation, followed by thorough washing with buffer containing 10 mM Tris-HCl (pH 7.4) for 10 min at room temperature (25 °C).

##### Hybridization and éMCA Measurements

Target hybridization was carried out via 3 h incubation by spotting 1 μL of 1 μM 21-mer target (fully complementary (wild type)-Fc, SNP_B-Fc (SNP positioned at bottom), SNP_T-Fc (SNP positioned at top), and SNP_M-Fc (SNP positioned at the middle) in 10 mM Tris buffer (pH 7.4) containing 0.5 M NaCl, onto the functionalized electrode surface. Following hybridization, the sensor was extensively washed with the hybridization buffer. The array was then covered with the microfluidics using the double adhesive gasket and was placed between two aluminum plates of the heating device and the temperature ramped. All electrochemical measurements were carried out using an Autolab model PGSTAT 12 potentiostat/galvanostat controlled with the General Purpose Electrochemical System (GPES) software (Eco Chemie B.V., The Netherlands) 64-channel potentiostat, which was connected to the multielectrode array through an in-house fabricated connector box. A classical reference electrode Ag/AgCl was used. All the potentials are recorded with respect to the reference electrode. Parallelized multiple SWV measurements were recorded throughout temperature ramping. The parameters employed in the SWV experiments were as follows: potential window between 0 and 0.7 V (vs Ag/AgCl), 10 mV step potential, 0.1 V modulation amplitude, and 25 Hz frequency.

##### Asymmetric Polymerase Chain Reaction and Amplicon Detection

Asymmetric PCR was carried out in two stages: first, 25 cycles of PCR with both primers, followed by 12 cycles with just forward primer. The first PCR was carried out using Fc-labeled forward primer (Fc-FwP) and phosphate-labeled reverse primer (phosphate RvP), in a T100 thermal cycler (Biorad) following the protocol: 95 °C for 2 min, followed by 25 cycles at 95 °C for 30 s, 58 °C for 30 s, and 72 °C for 30 s, with a final elongation step at 72 °C for 5 min. Each 25 μL of PCR reaction mixture contained 1 unit of DreamTaq and DreamTaq buffer 1X, both forward and reverse primers (Fc-FwP and phosphate RvP) at 0.2 μM, dNTPs at 200 μM, and 100 pM synthetic DNA as final concentrations. In the second step, amplification was carried out applying 95 °C for 2 min, followed by 12 cycles of 95 °C for 30 s, 58 °C for 30 s, and 72 °C for 30 s, followed by a final elongation step at 72 °C for 5 min. Each 50 μL of asymmetric PCR mixture contained the same as the PCR reaction mixture but double the concentration of Fc-FwP (0.4 μM) and no RvP, and using 5 μL of PCR product from the first step as template DNA. The PCR product was then incubated with lambda exonuclease to digest the strand elongated with the phosphate-labeled primer in any remaining duplex DNA. The lambda exonuclease digestion via the addition of 1 unit of lambda exonuclease enzyme and 1× exonuclease buffer followed by incubation at 37 °C for 2 h and a final denaturation of the enzyme at 80 °C for 10 min.

Amplification products were purified using Oligo Clean DNA and concentrator (Ecogen, Spain) and checked using agarose gel electrophoresis. The gel was made with ultralow pure agarose (2.6% w/v) in 1× Tris-Borate-EDTA buffer (TBE) and stained with GelRed nucleic acid stain. A mixture of 5 μL of PCR product with 4 μL of 6× loading buffer was loaded per gel well, electrophoresis was performed at 110 V for 30 min, and gels were visualized in a UV transilluminator at λ = 254 nm.

éMCA with the single-stranded PCR amplicons was carried out in two ways. In the first approach, ferrocene-labeled amplicon was hybridized to four different probes. Eight electrodes of an array were modified with diverse thiolated probes (four probes in duplicate), one of which is fully complementary to the wild-type (i.e., the amplicon), and each of the other three contained a single mismatch at the top, bottom, or middle of the probe. In the second format, one common thiolated probe was immobilized on all the electrodes, and four different PCR amplicons were used, where the part of the amplicon that hybridizes to the immobilized probe is fully complementary or contains a mismatch at the top, bottom, or middle of the hybridized duplex.

In both approaches, measurements were carried out in duplicate, and two electrodes were incubated with each asymmetric PCR generated product for 3 h. After washing the electrode for 5 min, the electrode array was placed between the heating plates, covered by the microfluidics, and the Peltier applied to start the temperature ramp and the ferrocene signal measured throughout the ramp.

For all electrochemical melting curve analyses, eight parallel melting curves on a single electrode array were carried out simultaneously.

##### Fluorescence Melting Curve Analysis

Melting curve analysis was carried out using a fluorescence spectrophotometer (CARY Eclipse, Varian), housed with a Peltier thermostated multicell holder. This Peltier accessory contains chambers for four cuvettes. Ten microliters of the DNA duplex to be analyzed was added to a cuvette containing 100 μL of Tris-HCl (pH 7.4) and 0.2 μL of 20X Eva Green intercalating fluorescent dye. Each test was carried out by increasing the temperature from 25 to 95 °C at a fixed ramping rate, and the fluorescence continually measured at λ = 530 nm and the first derivatives of the melting curve used to accurately determine the melting temperature.

## Results and Discussion

The resulting transfer functions are:

Heating plate 1:
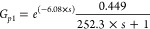


Heating plate 2:
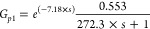


From the step response and simulated response obtained with Matlab, the step response of the transfer function is in concordance with the system behavior, allowing the use of this transfer function to model and study the closed-loop response of the PI controller in series with the system.

### PID tuning

The most common PID controllers were chosen to control the resolution of each of the heating blocks, because the proportional part improves the rising and settling time of the response, where the integrative part reduces the steady-state error; the derivative part is used to reduce both the overshoot and the change rate of the error. To control the step resolution, the correspondent *K*_p_ and *K*_i_ values were tuned to produce a system response with a fast-rising and settling time, and with the minimum or no overshoot (details in Figure S-1c and Figure S-2 for heating plate 2 (SI)). The best *K*_p_ and *K*_i_ parameters that produced the best response on both heating plates were 55.85 and 1.33, respectively. These variables allowed to have a rise time of 15.6 s, a settling time of 1 min and 50 s, and a 10.4% overshoot. The overshoot value in principle is unacceptable because with each change on the set point, the temperature would overpass this set point. However, when the heating experiments were carried out on the real system, the output never exceeded the set point. One possible explanation for this is that the simulation was made with a step response of 1 V, although the highest voltage change made by the PWM in order to increase the block temperature by 1 °C is 24.41 mV. This means that the input shift might not be that strong to produce such a response on the controller.

### Heating Test

The heating system is composed of two (3.6 × 4.3 × 0.1 cm^3^) aluminum sheets that have a BI BPC 680J resistor attached to each of two type k thermocouples. The first thermocouple, together with HANNA Hi 93531, works as a temperature reference for the second thermocouple, and it was used temporarily to make the corrections for the corresponding temperature of the second thermocouple. Furthermore, the second thermocouple is connected to an AD595 (Analog devices) that works both as a cold junction compensation system and as a thermocouple amplifier. The system output is 10 mV/°C. This second subsystem controls the reading of the heating block temperature. This signal is also used as the input for the PID controller (Figure S-1b, Figure S-2 for heating plate 2 (SI)). Some heating tests were performed to observe the plate’s behavior and to determine if the PWM was able to produce and to read 0.2 °C temperature changes. These tests were done on each of the heating blocks individually and enclosed in a plastic case for room temperature isolation, and from the results obtained, a significant difference between the reference and each reading system was observed.

This reason is related to the Hi 93531 device, which is a commercial thermometer that can be assumed to have an integrated correction circuit or makes a digital temperature correction. This suggests that some correction is required to increase the reading fidelity. After analyzing the heating tests data of each block, the difference between the reference and the temperature reading system output showed a linear behavior. There are two possible options to make the temperature correction: In the first option, a system capable of taking the block temperature as an input, to compare and correct each reading point by point and to give this signal as a feedback parameter to the PI controller, could be implemented. This would require a change in the design and the incorporation of a specialized and complicated circuit, which would have had increased the production costs and the system complexity. The other option was to incorporate a digital correction. By making a linear regression, a correcting equation was obtained and the Arduino controlled the point by point correction. After the corresponding digital temperature corrections were made, the stability and functionality for each of the resolutions were tested by making a heating test on each plate working in parallel. Figure. S-1d (SI) shows the system’s behavior for a heating test starting at 25 °C and finishing at 90 °C. Both heating systems are capable of producing a stable and correct output.

To validate the entire system functionality, a complete heating test was performed using a glass slide with a microfluidic section made out of PMMA glued on top of it. The glass slide and PMMA were the same as those used for electrode array and microfluidics fabrication, which are the more representative parts of the whole system addressing the highest contribution to thermal properties. The electrode part represents only 30 nm of sputtered Ti/TiO_2_ and 100 nm of sputtered gold and is highly thermally conductive so we considered that it does not further affect the heat transfer through the microfluidics. A thermocouple was attached to the glass and the complete setup was placed between heating plates. All data was recorded with the Arduino and the Hi 93531. Figure S-1e (SI) shows the system behavior and the glass slide temperature evolution throughout the entire test. It is important to recall that there is still some small difference between the reference and the thermocouples that needs to be corrected. Heating plate 1 has a temperature error of 3.37%, while heating plate 2 has a lower error of 1.78%. It is important to highlight that the highest temperature difference between the glass slide temperature and the set point is 1.4 °C. One possible explanation for this is that the heat transference from the plates to the glass is not completely efficient due to the difference from the thermal conductivity coefficients of the aluminum (205 W/mK) with the PMMA (0.17 W/mK) and the glass slide (1.05 W/mK).

### Electrochemical Melting Curve Analysis (éMCA) Measurements

Dithiolated probes were selected to strengthen the bond with the gold surface to avoid desorption of the capture probe from surface during the melting analysis. An alkyl-dithiol^24^ was used as a spacer to provide a more organized self-assembled monolayer, avoiding steric hindrance and facilitating accessibility of the target for efficient hybridization with the ferrocene-labeled target. By monitoring the decrease of the oxidation peak of the ferrocene in the consecutive square wave voltammograms (SWV), the melting curve can be constructed and melting temperature (*T*_m_) calculated by applying the first derivative.

As a control to ensure that the changes in the electrochemical signal were solely attributed to the thermally induced denaturation of the surface-tethered duplex, the stability of the ferrocene signal with increasing temperature was evaluated. A thiolated oligo functionalized with ferrocene was chemisorbed onto the surface of the gold electrode array and subjected to the temperature ramp employed in the melting curve analysis, and as can be seen in Figure S-3 (SI), the observed voltammetric signal was stable, thus demonstrating that any decrease in the signal was not due to probe desorption or instability of the ferrocene label at elevated temperatures.

**Figure 4:**
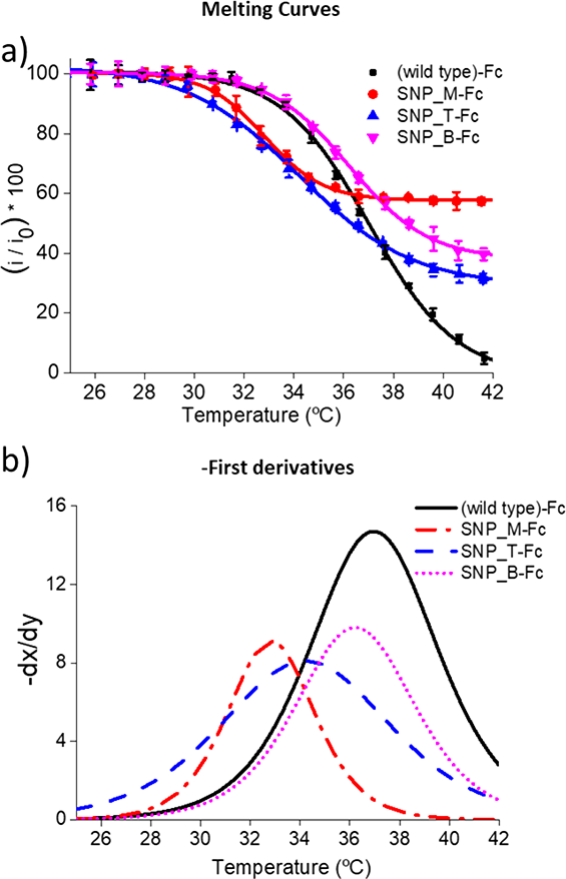
(a) Melting curves and (b) the corresponding first derivatives obtained for four different 21-mer synthetic targets hybridized to a common surface tethered capture probe, with each of the targets being the fully complementary wild-type ((wild type)-Fc) or containing a single mismatch at middle (SNP_M-Fc), top (SNP_T-Fc), or bottom (SNP_B-Fc) of the sequence. All measurements were carried out in triplicate.

Using the 1 °C/step temperature ramp, the melting temperatures of the duplexes formed between a common probe with four different ferrocene labeled 21-mer targets, one of which was fully complementary (wild-type), while the other contained a mismatch that was positioned at the top, bottom, or middle of the labeled oligo. Square wave voltammetry measured the peak attributed to ferrocene throughout the temperature ramp, and the measured signal was normalized to the signal obtained prior to initiation of the melt, and for a clear determination of the melting temperature first-derivative plots were used (Figure 4). As can be seen in Figure 4, where the melting curves and first derivatives of the melting curves were obtained using the 1 °C/step, the highest melting temperature, as expected, is for the fully complementary duplex. The largest difference in the melting temperature is achieved when the single mismatch is positioned in the center of the probe, but in all cases, a clear difference in melting temperature can be observed.

Once the proof-of-concept of electrochemical melting curve analysis had been demonstrated with the short 21-mer fully complementary target, the methodology was applied to a full-length 124-mer PCR amplicon. Due to the intramolecular collision, followed by rapid zipping nature of DNA hybridization, the kinetics of hybridization of the 21-mer with the immobilized probe can be expected to be faster as compared to the 124-mer single-stranded PCR amplicon. However, a lengthy hybridization time of 3 h was used to ensure full hybridization of the 124-mer. It can also be postulated that for the 21-mer target hybridized to the surface primer the ferrocene moiety would be confined in the organic layer forming a more regular pattern as compared to the 124-mer PCR amplicon, thus facilitating a slightly higher electrochemical signal.

**Figure 5:**
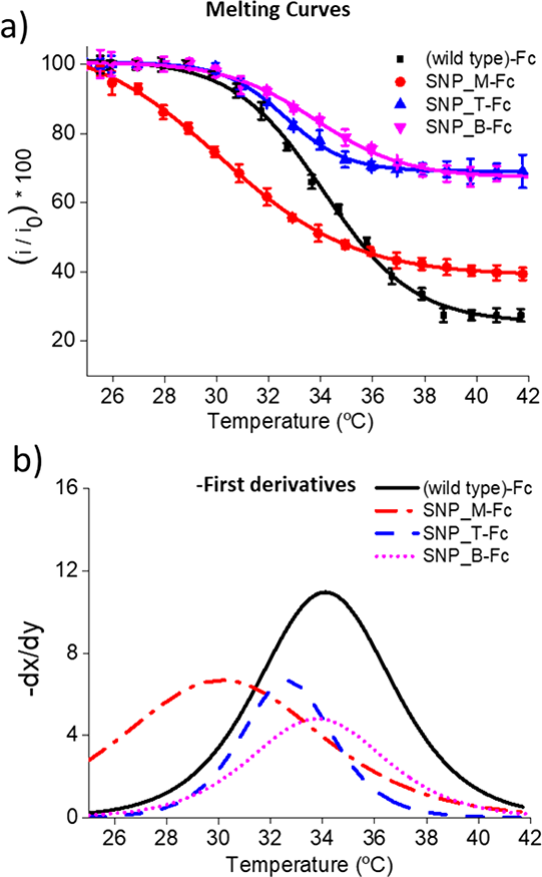
Melting curves and the corresponding first derivatives obtained for a common 124-mer amplicon hybridized to four different surface tethered capture probes, with each of the probes being the fully complementary wild-type ((wild type)-Fc) or containing a single mismatch at middle (SNP_M-Fc), top (SNP_T-Fc) or bottom (SNP_B-Fc) of the sequence. All measurements were carried out in triplicate.

**Figure 6:**
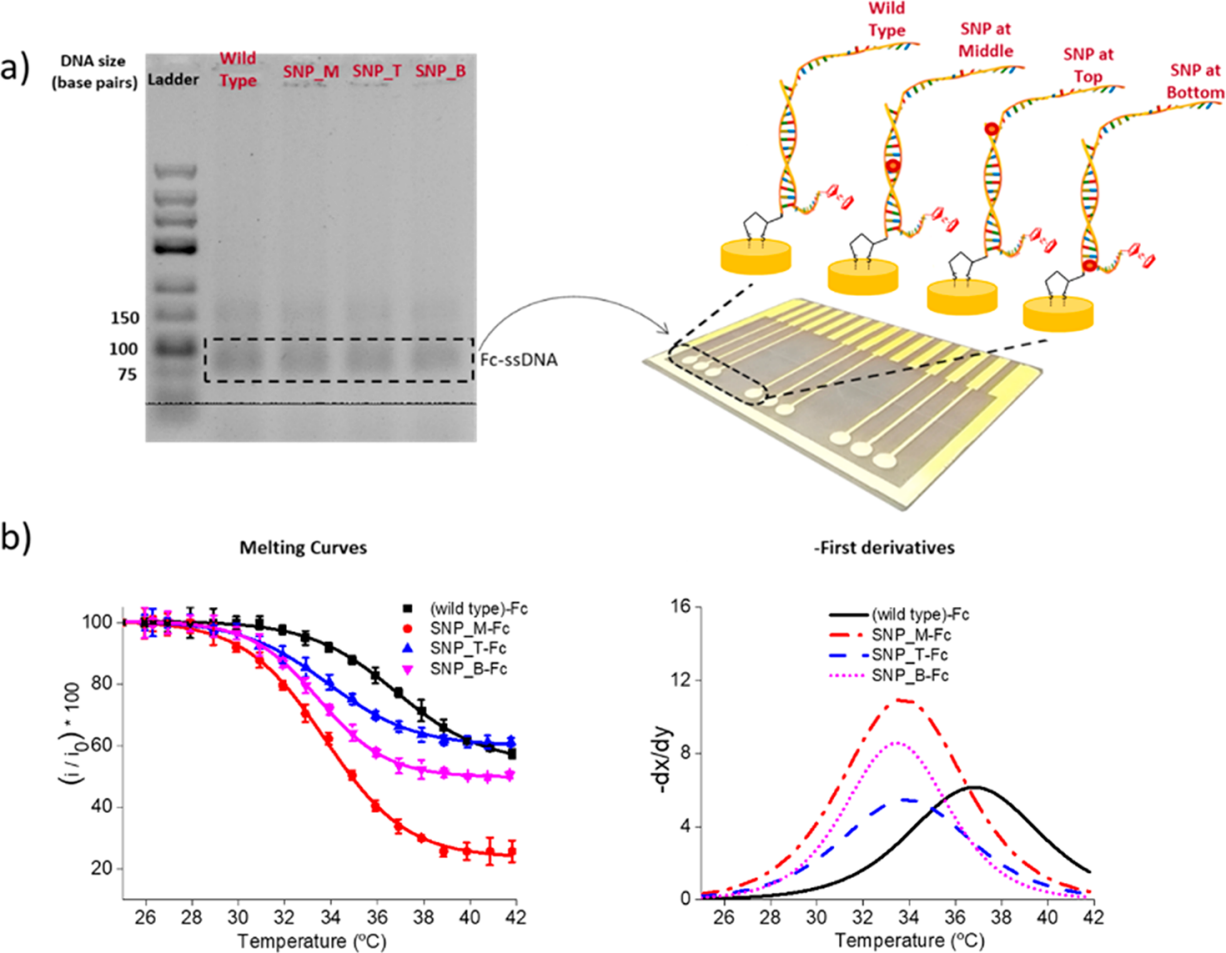
(a) Agarose gel electrophoresis analysis of each of the ssDNA 124-mer amplicons obtained after asymmetric PCR amplification, with the amplicons designed to be fully complementary (wild-type) or to contain a single mismatch designed to be at the top, bottom, or middle of the hybridized duplex. (b) Melting curves obtained for the four different asymmetric PCR targets and the corresponding first derivatives. All measurements were carried out in triplicate.

The assay for the analysis of the 124-mer amplicons was thus evaluated using two different formats, one using one common PCR amplicon and four different immobilized probes (Figure 5) and the second using a common surface-tethered probe and four different single-stranded PCR amplicons (differing by one mismatch and the polymorphic site) (Figure 6). Both approaches were used to evaluate the effect of the position of the SNP site to be interrogated in the hybridized duplex and to elucidate if a better discrimination between fully complementary (wild-type) and single mismatch (presence of non-wild-type SNP), can be achieved by optimization of this position. In both cases, single-stranded DNA was generated by a combination of asymmetric PCR and exonuclease digestion and checked using gel electrophoresis (Figure S-4 (SI)). The example for the second approach is given in Figure 6a, where the gel electrophoresis of ssDNA after the PCR (DNA) and exonuclease digestion (ssDNA) are shown.

The melting curves and first derivatives for the first approach can be seen in Figure 5 and Figure 6, and it is clear that the highest melting temperature is obtained with the fully complementary wild-type, and again similar to the 21-mer, the biggest difference between the wild-type and single mismatch is obtained when this mismatch is positioned in the center of the probe.

The results obtained using electrochemical melting analysis were then compared with those determined using fluorescence melting curve analysis to see if similar trends were obtained. It should be noted that similar melting temperatures are not expected, as it is known that the melting temperature of surface tethered DNA duplexes are around 10 °C lower than that obtained in solution.^25^ As can be seen in Table 1, very similar trends were observed, with the biggest difference in melting temperature between fully complementary and single mismatch containing is achieved when the mismatch is in the middle of the hybridized duplex.

**Table 1:** *T*_m_ Calculated from Different Melting Curves

	melting temperature (*T*_m_/°C) mean value *T*_m_ ± standard deviation (*n* = 3)				
	21-mer target		124-mer target Asym-PCR product		
SNP position	éMCA	fluorescence MCA	éMCA 4 Fc-targets/1 capture probe	éMCA 1 Fc-target/4 capture probes	fluorescence MCA 1 Fc-target/4 capture probes
Fully complementary	37.0 ± 0.9	46.6 ± 3.3	36.3 ± 1.2	34.1 ± 0.5	46.5 ± 1.2
Mismatch in middle	32.7 ± 0.6	24.6 ± 1.7	32.7 ± 1.1	30.0 ± 0.7	31.4 ± 1.6
Mismatch at top	34.9 ± 0.1	30.6 ± 1.9	34.0 ± 0.7	32.6 ± 1.2	34.2 ± 0.1
Mismatch at bottom	36.2 ± 1.5	32.4 ± 1.3	35.1 ± 0.5	33.8 ± 0.9	34.5 ± 0.5

The results obtained are in agreement with multiple previous studies, where terminal mismatches in short duplexes are known to have less effect than internal mismatches.^26^ Mismatches near the center of the probe have been reported to have a stronger destabilizing effect than mismatches close to either end, both for hybridizations in solution^27^ and for microarray hybridizations,^28,29^ and this difference in destabilization has been observed frequently, and used in applications such as SNP detection.^30,31^

Letowski et al. evaluated the effect of probe size, mismatch position, as well as the number of mismatches and concluded that mismatches at the ends of a duplex have markedly lower effects as compared to a middle mismatch.^32^ Lievens et al. used chemiluminescence to explore the influence of mismatch positions at regular intervals of positions (i.e., 1, 5, 10, 15, and 20) in different 20-mer sequences and varying types of mismatch at these positions and reported that mismatches at extreme positions are least distinguishable and are independent of the type of sequence.^33^ In agreement with these studies, Naiser et al. found that thermodynamically a middle mismatch was found to be less stable in a 16-mer sequence.^34^ Rennie et al. carried out a large-scale investigation of microarray hybridizations to murine probes with known sequence mismatches, demonstrating that the effect of mismatches is strongly position-dependent, being stronger for mismatches near the center of the probe than for those at the ends.^35^ El-Yazbi et al. used a terbium(III) luminescent probe to study the effect of mismatch position and found that when comparing strands with only one mismatch, the oligonucleotide with a mismatch located at the center of the oligomer exhibited the most Tb3 luminescence enhancement due to duplex instability.^36^

In summary, we have developed a semiautomated device for the detection of single base mismatches using electrochemical melting curve analysis, where an in-house Peltier device was used to apply a controlled heating ramp to an electrode array. The optimum position of the SNP site to be interrogated in relation to the immobilized probe has been elucidated. While multiplexed detection of SNPs was not demonstrated, multiple different duplexes were simultaneously subjected to melting curve analysis (in each array, eight electrodes with duplicates for each allele and one control electrode), and future work will focus on multiplexed detection of SNPs, first using low-density electrode arrays and then moving to arrays with a high number of individual electrodes.

## Conclusions

We detailed an in-house-fabricated device for multiplexed electrochemical melting curve analysis. The system consists of temperature controller (homemade Peltier) integrated with an electrode array housed within a microfluidic device, with multiplexed electrochemical detection. The platform was primarily demonstrated with a short DNA duplex, and the effect of the position of the mismatch interrogated. The platform was then extended to duplexes with a short surface-tethered DNA probe and a 124-mer single-stranded asymmetric PCR amplicon. Again, with the aim of elucidating the best position for mismatch discrimination, two different approaches were evaluated—one where four different probes were mismatched at different positions and a single amplicon, and the other with a single probe and four different amplicons. In all cases, the optimum position for maximum destabilization of the surface-tethered DNA duplex and consequent optimal discrimination between fully complementary and single mismatch containing was observed with the mismatch site at the center of the hybridized DNA duplex. The developed device is semiautomated, capable of multiplexed detection, relatively rapid, cost-effective, and easy-to-use, and future work will focus on detection of disease-associated SNPs in blood samples, and moving from PCR to isothermal amplification.

## Supporting Information Available

Oligonucleotides used in this study; Examples of the system’s behavior for a heating test starting at 25 °C and finishing at 90 °C demonstrating the stability of the output; Demonstration of the stability of surface chemistry vs temperature and repetitive potential cycling; Agarose gel electrophoresis after each step of the single-stranded redox labeled PCR amplicon generation based on a combination of asymmetric PCR and Lambda exonuclease digestion (PDF)

## Abbreviations

éMCA: electrochemical melting curve analysis
*T*_m_: melting temperature
SNP: single nucleotide polymorphism
SWV: square wave voltammetry
