# Supplementary material for: Low-Cost Platform for Multiplexed Electrochemical Melting Curve Analysis
Source: ACS Meas Sci Au. 2021 Nov 22;2(2):147–56. doi: 10.1021/acsmeasuresciau.1c00044 (PMC9031717; doi:10.1021/acsmeasuresciau.1c00044)
Supplement: Supplementary file 1 — tg1c00044_si_001.pdf [file tg1c00044_si_001.pdf]

Supporting Information

Low cost platform for multiplexed electrochemical melting curve analysis

Nassif Chahin[a], Santiago Escobar-Nassar,[a,b] Johann Osma[b], Abdulaziz S. Bashammakh[c], Abdulrahman O. Alyoubi[c], Mayreli Ortiz[a]*, Ciara K. O'Sullivan[a,d]*

[a] *Department d'Enginyeria Química, Universitat Rovira i Virgili, Avinguda Països Catalans 26, 43007 Tarragona, Spain.*

[b]*Faculty of Engineering, Universidad de los Andes, Colombia*

[c]*Department of Chemistry, Faculty of Science, King Abdulaziz University, P.O. Box 80203, 21589 Jeddah, Kingdom of Saudi Arabia*

[d]*ICREA, Passeig Lluis Companys 23, 08010 Barcelona, Spain*

Email: mayreli.ortiz@urv.cat,ciara.osullivan@urv.cat, ckosulli@gmail.com

Table of Contents

Table S-1. Oligonucleotides used for the melting curve analysis in this study: capture probes, 21-mer targets, 124-mer targets, and the primers used to generate the ferrocene-targets by asymmetric-PCR. **2**

Figure S-1 (a) Step response and simulated transfer function of the heating plate 1 obtained with Matlab; (b) Modelled closed loop response of the PID controller in series with the heating block; (c) Modelled system of the PID controller in series with the transfer function plate that simulates the behaviour of the heating plate 1; (d) Example of the system's behaviour for a heating test starting at 25°C and finishing at 90°C. Both heating plates produced a stable output; (e) The heating ramp of the complete system with a glass slide – PMMA template in the middle of the heating plates. **3**

Figure S-2 (a) Step response and simulated transfer function of the heating plate 2 obtained with Matlab; (b) Modelled closed loop response of the PID controller in series with the heating block; (c) Modelled system of the PID controller in series with the transfer function plate that simulates the behaviour of the heating plate 2. **4**

Figure S-3 (a) Repetitive SWVs of double functionalized Fc-DNA-SH on gold surface with temperature ramping; (b) Repetitive SWVs of double functionalized Fc-DNA-SH on gold surface during 1 h at 25°C. **4**

Figure S-4 Agarose gel electrophoresis after each step of the single-stranded redox labelled PCR amplicon generation based on a combination of asymmetric PCR and Lambda exonuclease digestion. **5**

Table S-1. Oligonucleotides used for the melting curve analysis in this study: capture probes, 21-mer targets, 124-mer targets, and the primers used to generate the ferrocene-targets by asymmetric-PCR. (The regions of the target sequences complementary to the capture probes are underlined)

Oligonucleotides	Sequences (5\` to 3\`)
**Approach 1:** 21-mer targets	
**Wild type (Full complementary)**	5′-Fc-CGAAGTGTGAACTAGTCCCAC-3′
**SNP at the top**	5′-Fc- **A**GAAGTGTGAACTAGTCCCAC-3′
**SNP at the middle**	5′-Fc-CGAAGTGTGAA**A**TAGTCCCAC-3′
**SNP at the bottom**	5′-Fc-CGAAGTGTGAACTAGTCCCA**A**-3′
**Approach 2 (**4 Fc-targets / 1 capture probe**) and Approach 3 (**1 Fc-target / 4 capture probes**): 124-mer targets**	
**No SNP (Full complementary)**	5\`-AGCTCCAGAAGATAAATTACAGGCGAAGTGTGAACTAGTCCCACCACCTTAATTTCACTGTGTGTTAACACTTGTAAAGAACCTGCATAATGTGTGTATCTTACAACTAGGATACTATGACCCC-3\`
**SNP at the top**	5\`-AGCTCCAGAAGATAAATTACAGGCGAAGTGTGAACTAGTCCCA**A**CACCTTAATTTCACTGTGTGTTAACACTTGTAAAGAACCTGCATAATGTGTGTATCTTACAACTAGGATACTATGACCCC-3\`
**SNP at the middle**	5′-AGCTCCAGAAGATAAATTACAGGCGAAGTGTGAA**A**TAGTCCCACCACCTTAATTTCACTGTGTGTTAACACTTGTAAAGAACCTGCATAATGTGTGTATCTTACAACTAGGATACTATGACCCC-3\`
**SNP at the bottom**	5′-AGCTCCAGAAGATAAATTACAGG**A**GAAGTGTGAACTAGTCCCACCACCTTAATTTCACTGTGTGTTAACACTTGTAAAGAACCTGCATAATGTGTGTATCTTACAACTAGGATACTATGACCCC-3\`
**PCR primers for Ferrocene incorporation**	Fc-Forward primer: 5′-Fc-AGCTCCAGAAGATAAATTACAGG-3\`<br>Reverse primer: 5′-pho-GGGGTCATAGTATCCTAGTTG-3\`
**Capture probes for all approaches**	
**Full complementary to the target (thiol 3′-)**	5′-GTGGGACTAGTTCACACTTCGTTT-3′-Thiocticacid
**SNP at the top**	5′-**A**TGGGACTAGTTCACACTTCGTTT-3′-Thiocticacid
**SNP at the middle**	5′-GTGGGACTA**A**TTCACACTTCGTTT-3′-Thiocticacid
**SNP at the bottom**	5′-GTGGGACTAGTTCACACTTC**A**TTT-3′-Thiocticacid
**Surface control**	
**Fc-DNA-thiol**	5′-Fc-GTGGGACTAGTTCACACTTCGTTT-3′-C6-Thiol

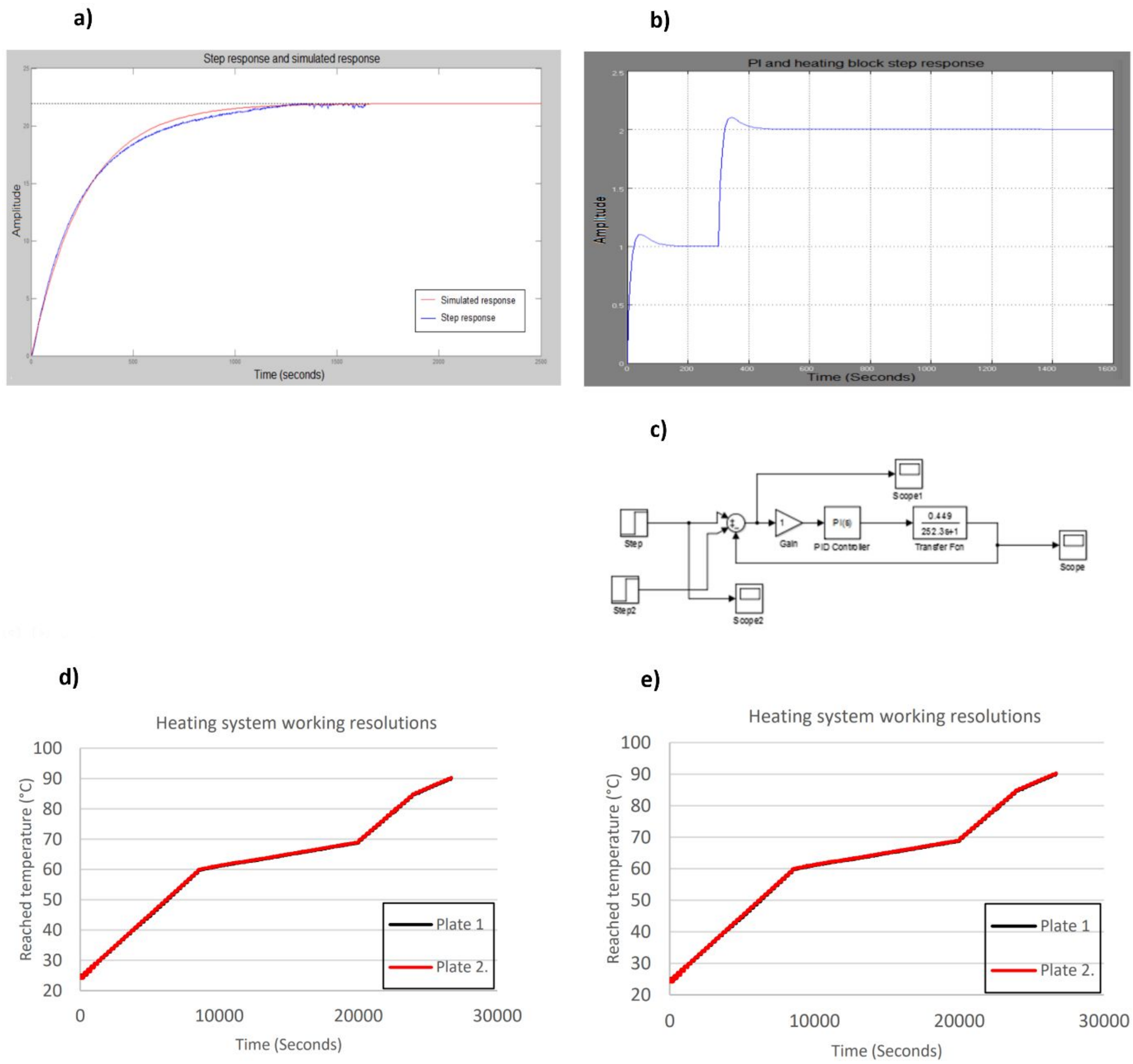

Figure S-1 (a) Step response and simulated transfer function of the heating plate 1 obtained with Matlab; (b) Modelled closed loop response of the PID controller in series with the heating block; (c) Modelled system of the PID controller in series with the transfer function plate that simulates the behaviour of the heating plate 1; (d) Example of the system's behaviour for a heating test starting at 25°C and finishing at 90°C. Both heating plates produced a stable output; (e) The heating ramp of the complete system with a glass slide – PMMA template in the middle of the heating plates.

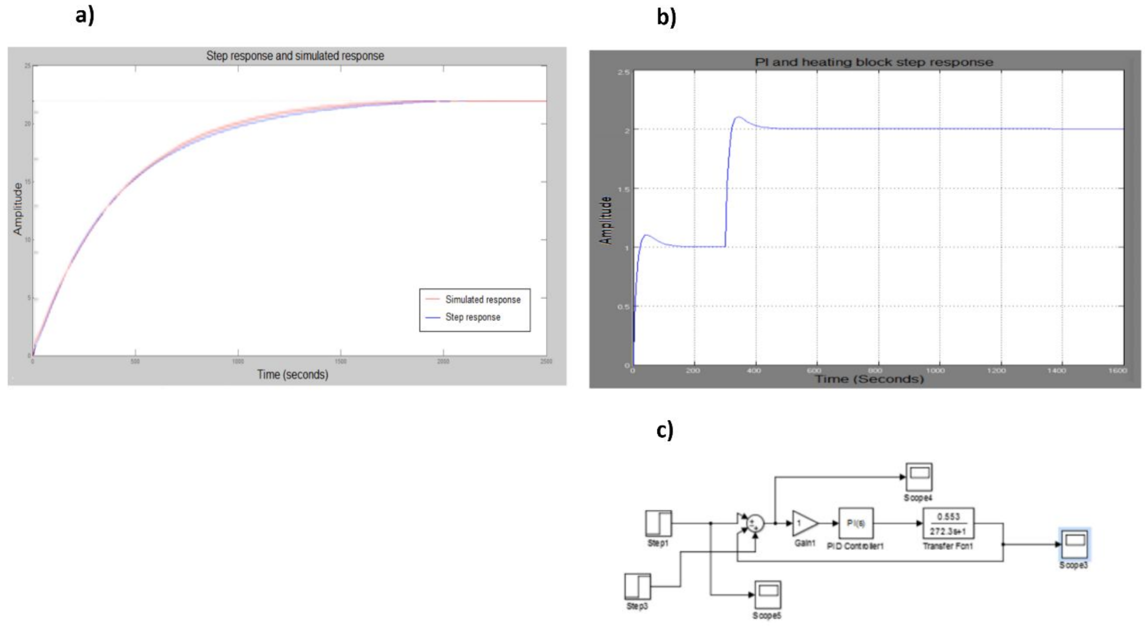

Figure S-2 (a) Step response and simulated transfer function of the heating plate 2 obtained with Matlab; (b) Modelled closed loop response of the PID controller in series with the heating block; (c) Modelled system of the PID controller in series with the transfer function plate that simulates the behaviour of the heating plate 2.

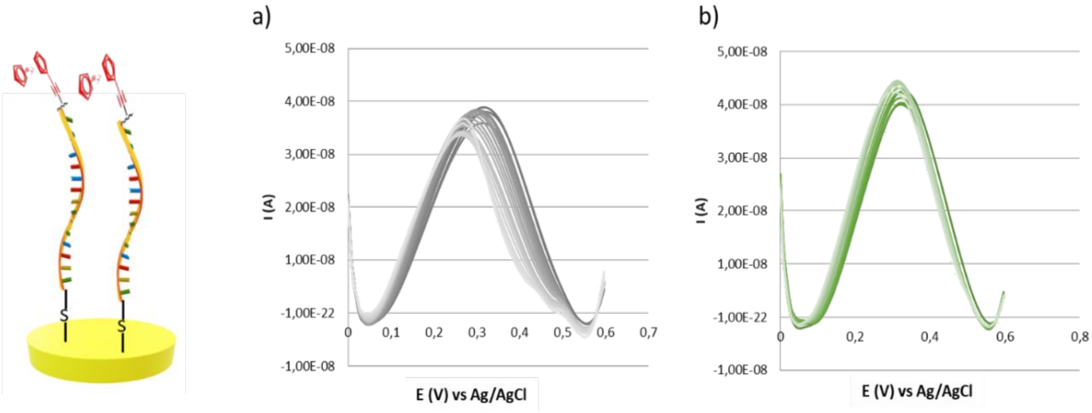

Figure S-3 (a) Repetitive SWVs of double functionalized Fc-DNA-SH on gold surface with temperature ramping; (b) Repetitive SWVs of double functionalized Fc-DNA-SH on gold surface during 1 h at 25ºC.

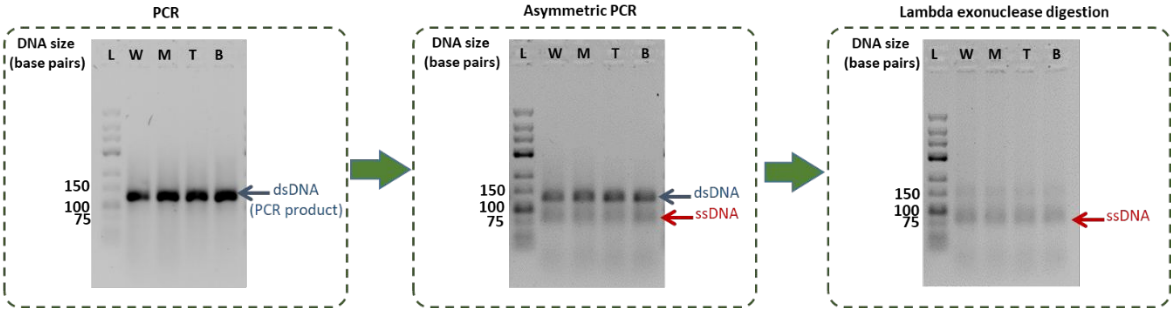

Figure S-4 Agarose gel electrophoresis after each step of the single-stranded redox labelled PCR amplicon generation based on a combination of asymmetric PCR and Lambda exonuclease digestion.
